# Effect of Sodium Arsenite on the Expression of Antioxidant Genes (*SOD2* and *CAT*) in MCF-7 and Jurkat Cell Lines

**Published:** 2017-02

**Authors:** Leila FALLAHZADEH-ABARGHOOEI, Maryam SAMADAEI-GHADIKOLAIE, Iraj SAADAT, Mostafa SAADAT

**Affiliations:** Dept. of Biology, College of Sciences, Shiraz University, Shiraz, Iran

**Keywords:** Catalase, mRNA, N-acetyl-cysteine, *SOD2*, Sodium arsenite, MCF-7 cells, Jurkat cells

## Abstract

**Background:**

Sodium arsenite (NaAsO2) has potent cytotoxic activity in human cancer cells. Oxidative stress has been suggested as a mechanism for arsenic-induced carcinogenesis. The purpose of the present study was to evaluate the alteration of mRNA levels of catalase (*CAT*) and superoxide dismutase 2 (*SOD2*) in MCF-7 and Jurkat cells after exposure to NaAsO_2_.

**Methods:**

Methylthiazol tetrazolium (MTT) viability assay was performed to evaluate cytotoxicity of NaAsO_2_ in MCF-7 and Jurkat cells. For evaluating the expression levels of the *CAT* and *SOD2*, we used two concentrations of NaAsO_2_ (5 and 15 μM), lower than the concentrations at which 50% of cell viability were lost. The cells were treated with co-treatment of NaAsO_2_ (15 μM) and N-acetyl-cysteine (NAC; 5 μM) in the media for 24 h. The control cells were maintained in sodium arsenite free growth medium. The experiments were done triplicate. Using quantitative real-time PCR, the expression levels of *CAT* and *SOD2* were quantified. One sample student’s *t* test was performed for comparisons of mRNA levels between treatment groups and their corresponding untreated control cells.

**Results:**

*CAT* mRNA level decreased significantly in both cell lines following exposure to NaAsO_2_ (*P*<0.05). Expression levels of *SOD2* decreased in Jurkat cells and increased in MCF-7 cells after treatment with NaAsO_2_ (*P*<0.05).

**Conclusion:**

After cells exposure to NaAsO_2_, *CAT* mRNA level decreased in both examined cell lines but the alterations of *SOD2* mRNA level is cell specific. The NAC modulated the NaAsO_2_ associated alterations of *CAT* and *SOD2* mRNA levels, therefore, the NaAsO_2_ might act through inducing reactive oxygen species.

## Introduction

Arsenic is a major global health concern due to its adverse health effects (1, 2). Arsenic compounds are widely distributed naturally in soil, water, and food (3). Reactive oxygen species (ROS) such as superoxide anion (O_2_^·−^) and hydrogen peroxide (H_2_O_2_) are produced in various cellular systems exposed to arsenite (4–7).

Oxidative stress has been involved in mediating many deleterious effects of arsenic. A variety of oxidative stress biomarkers (including DNA damage, lipid peroxidation, redox enzyme activity, decreased antioxidant defense levels and changes in gene expression) have been suggested induced by arsenic (7–12). Previously, in HeLa cells exposed to NaAsO_2_ (2μM), the expression levels of superoxide dismutase 2 (*SOD2*), glutathione S-transferase T1 (*GSTT1*) and glutathione S-transferase M1 (*GSTM1*) decreased compared to untreated cells, although this change did not reach statistical significance (13).

Exposure to arsenic reduces antioxidant levels (14). Catalase (CAT; EC: 1.11.1.6, OMIM: 115500) and SOD2 (EC: 1.15.1.1. OMIM: 147460) are antioxidant enzymes that metabolize ROS.

Functional polymorphisms of *CAT* (15–18) and *SOD2* (19–21) are associated with susceptibility to several multifactorial traits which oxidative stress are involved in their pathogenesis. Oxidative stress in human SH-SY5Y cells was induced by treatment with methadone and morphine and subsequently the mRNA levels of *CAT* showed significant alterations (22, 23). Sodium arsenite is associated with oxidative stress (24, 25). Based on recent reports, NaAsO2 has potent cytotoxic activity in human cancer cells in vitro and in vivo (26).

In the present study, we used two human cell lines, MCF-7 (breast cancer) and Jurkat (T cell leukemia) cell lines which represent cancer models, in order to investigate the alterations of *CAT* and *SOD2* mRNA levels in the cells exposed to sodium arsenite.

## Materials and Methods

### Cell Culture and Treatment

The present study had an experimental design. MCF-7 (NCBI C135; a breast cancer cell line) and Jurkat E6.1 (NCBI C121; a human T lymphoblastoid cell line) were obtained from National Cell Bank of Iran (the Pasteur Institute of Iran, Tehran). The MCF-7 and Jurkat cell lines were cultured in RPMI-1640 medium supplemented with 10% fetal bovine serum and 0.1 mg/ml streptomycin and 1000 IU/ml penicillin (Gibco). Cells were grown at 37 °C in a humidified atmosphere of 5% CO_2_ in air. The cells were treated with sodium arsenite (final concentrations 5 and 15 μM). Moreover, the cells were treated with co-treatment of sodium arsenite (final concentration 15 μM) and N-acetyl-cysteine (NAC; C_5_H_9_-NO_3_S; final concentration 5 μM) in the media for 24 h. The NAC is generally being used as an antioxidant. The control cells were maintained in sodium arsenite free growth medium. The experiments were done triplicate.

### MTT assay

The 3-(4, 5-dimethylthiazol-2-yl) -2, 5-diphenyl tetrazolium bromide (MTT) viability assay was performed to evaluate cytotoxicity of NaAsO2 in MCF-7 and Jurkat cells. Briefly, cells were seeded at a density of 2×10^5^ cells/well on 96-well plate. After attachment, various concentrations of sodium arsenite (10, 20, 40, 80 μM for MCF-7 cells; 0.5, 1, 5, 10, 50, and 100 μM for Jurkat cells) were added for 24 h. Then, MTT solution was added to each well and incubated for 4 h at 37 °C. Formazan was solubilized by adding 100μl of 10% SDS containing 0.01 M HCl to each well and incubated at 37 °C for overnight. Finally, optical density was measured using ELISA reader at wavelength 570 nm. The absorbance correlates linearly to the number of living cells in the culture. Three wells were used for each concentration. The experiment was repeated three times. We used 50% cell viability loss as a cytotoxicity index that reduces the cell number to 50% compared to untreated control cells.

### RNA extraction, cDNA synthesis, and Real-time PCR

Total RNA was purified from the treated cells with RNX-plus™ solution (Cinnagen, Iran) following the manufacturer’s instruction. The quantity of the RNA was measured with spectrophotometer at a wavelength of 260 nm (A_260_). The purity of extracted total RNA was determined by the A_260_/A_280_ ratio (for our samples it was 1.8–2.1). RNA was reverse transcribed with MuLV reverse transcriptase and oligo-d (T) primers (Takara¸ Japan). Quantitative real-time PCR was conducted using a Rotor-gene 6000 real-time PCR system (Corbett Life Science) and SYBR Green master mix (Takara¸ Japan). The sequences of primer pairs used for *β-actin*, *CAT* and *SOD2* were mentioned in Table 1.

**Table 1:** Primer sets used for real-time PCR

**Genes**		**Sequences**
*β-actin*	F	5′-CGAGCACAGAGCCTCGCCTT-3′
	R	5′-ACATGCCGGAGCCGTTGTCG-3′
*CAT*	F	5′-GGATCCCGCCAGCGACCAGA-3′
	R	5′-ACCCACGAGGGTCCCGAACTG-3′
*SOD2*	F	5′-CTGCTGGGGATTGATGTGTGG-3′
	R	5′-TGCAAGCCATGTATCTTTCAGT-3

PCR reactions were completed in 20 μl final volume containing 1μl of each primer, 4 μl cDNA (corresponding 20 ng) and 10 μl SYBR green master mix reagent (Takar, Japan). PCR conditions consisted of initial denaturation at 95 °C for 30 sec, followed by 40 cycles of denaturation at 95 °C for 10 sec, annealing at 60 °C for 15 sec and extension at 72 °C for 20 sec. Specificity of PCR products was tested according to the dissociation curves. Relative differences in gene expression between groups were expressed using cycle time (Ct) values. These Ct values were first normalized with that of *β-actin* in the same sample and then expressed as fold with control set to 1.0. Relative values of transcripts were calculated using the equation: 2^−ΔΔCt^, where ΔCt is equal to the difference in threshold cycles for target and reference.

Shiraz University Ethics Committee approved this study. The work has been carried out in accordance with Code of Ethics of the world Medical Association (Declaration of Helsinki) for experiments in humans and animals.

### Statistical analysis

Data were expressed as mean ± SD. One sample Student’s *t* test was performed for comparisons of mRNA levels between treatment groups and their corresponding untreated control cells. The independent samples *t*-test was applied to detect difference of the expression level between cells exposed to NaAsO_2_ and treated by NaAsO_2_ plus NAC.

Statistical analysis was performed using the Statistical Package for Social Sciences (SPSS Inc., Chicago, IL, USA) (ver. 11.5). The *P*-values less than 0.05 were considered statistically significant.

## Results

**Fig. 1:**
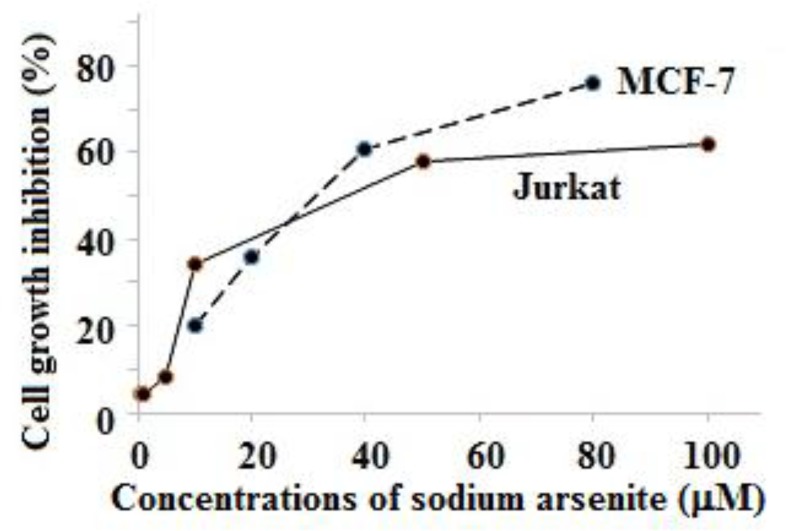
Relationship between sodium arsenite concentrations and percent of cell growth inhibition

In order to study the cytotoxicity of sodium arsenite on MCF-7 and Jurkat cells, the cells were exposed to different concentrations of NaAsO_2_. In MCF-7 cells, the inhibitory effects were observed after incubation with 10, 20, 40 and 80 μM NaAsO_2_ as reducing cell growth by 20, 36, 61, 76 and 88%, respectively. In Jurkat cells, after incubation with 0.5, 1, 5, 10, 50, and 100 μM NaAsO_2_, cell growth reduced by 4, 4, 8, 34, 58 and 62%, respectively. Fig. 1 shows that cell growth inhibition significantly increased as function of sodium arsenite concentration (*P*<0.05). The 50% of cell viability loss for MCF-7 and Jurkat cell lines were observed at 35 and 45 μM of NaAsO_2_, respectively. For evaluating the expression levels of the *CAT* and *SOD2*, we used two concentrations of NaAsO_2_ (5 and 15 μM), lower than the concentrations at which 50% of cell viability were lost.

Table 2 shows the effect of sodium arsenite on the mRNA levels of *CAT* in MCF-7 and Jurkat cells. When MCF-7 and Jurkat cells were treated with sodium arsenite (5, 15 μM), *CAT* mRNA level decreased significantly as compared with untreated control cultures in a dose-dependent fashion (*P*<0.05). The mRNA level of *CAT* in MCF-7 and Jurkat cells exposed to NaAsO_2_ plus NAC increased significantly as compared with the cells exposed to NaAsO_2_ (*P*<0.05).

**Table 2:** Expression level of *CAT* in MCF-7 and Jurkat cells after exposed to NaAsO_2_ and NaAsO_2_ plus N-acetylcysteine (NAC)

**Treatment**	Mean*	**SD**	**t**	**df**	***P*-value**
**MCF-7 cell**					
NaAsO_2_ (5 μM)	0.53	0.03	19.69	2	0.003
NaAsO_2_ (15 μM)	0.26	0.04	24.86	2	0.002
NaAsO_2_ (15 μM) + NAC	0.43	0.04	4.26	4	0.013
**Jurkat cell**					
NaAsO_2_ (5 μM)	0.53	0.16	4.08	2	0.055
NaAsO_2_ (15 μM)	0.45	0.16	4.77	2	0.041
NaAsO_2_ (15 μM) + NAC	0.96	0.11	3.67	4	0.021

* The fold change of the expressions compared to control untreated cells

Table 3 shows the effect of NaAsO_2_ on the *SOD2* mRNA levels in MCF-7 and Jurkat cells. In MCF-7 cells, *SOD2* mRNA level increased significantly in cells exposed to NaAsO_2_ as compared with untreated control cells (*P*<0.05). Reduction of mRNA level of *SOD2* in MCF-7 cells treated with NaAsO_2_ plus NAC was observed as compared to the cells exposed to NaAsO_2_ (*P*<0.05). Sodium arsenite caused significant decrease in expression of *SOD2*. In Jurkat cells, combination treatment of NaAsO_2_ plus NAC was associated with an elevation of mRNA level of *SOD2* when compared with the cells treated by NaAsO_2_.

**Table 3:** Expression level of *SOD2* in MCF-7 and Jurkat cells after exposed to NaAsO_2_ and NaAsO_2_ plus N-acetylcysteine (NAC)

**Treatment**	Mean*	**SD**	***t***	**df**	***P*-value**
**MCF-7 cell**					
NaAsO_2_ (5 μM)	1.60	0.04	28.82	2	0.001
NaAsO_2_ (15 μM)	1.87	0.05	27.25	2	0.001
NaAsO_2_ (15 μM) + NAC	0.89	0.06	19.91	4	<0.001
**Jurkat cell**					
NaAsO_2_ (5 μM)	0.38	0.13	6.67	2	0.022
NaAsO_2_ (15 μM)	0.28	0.02	41.57	2	0.001
NaAsO_2_ (15 μM) + NAC	0.56	0.10	3.87	4	0.018

* The fold change of the expressions compared to control untreated cells

## Discussion

Short time arsenic exposure (24h) causes a significant decrease in expression of *CAT* in the both study cell lines, whereas, the mRNA levels of *SOD2* in MCF-7 and Jurkat cells, exposed to NaAsO_2_ were significantly decreased and increased, respectively (Table 2, 3).

The mRNA and protein levels as well as enzyme activity of CAT decreased significantly in human keratinocyte (HaCaT) cell line exposed to sodium arsenite (27). The CAT enzyme activity decreased in rat livers after arsenic administration (28). These findings are consistent with our present results (Table 2).

The *SOD2* mRNA level increased in treated umbilical vein endothelial cells (HUVECs) with a low concentration of NaAsO2 (29). Down-regulation of *SOD2* in skin lesions which treated by arsenic was reported (30). In the present study, in treated MCF-7 and Jurkat cells with NaAsO_2_, the mRNA levels of *SOD2* were significantly decreased and increased, respectively (Table 3). Response of cells to NaAsO_2_ treatment is dependent on the type of cells. *SOD2* have several genetic polymorphisms. These genetic variations might be involved in mRNA expression and play a critical role in human phenotypic diversity (31). Genomic variations in the *SOD2* gene involved in different response of the *SOD2* mRNA level in exposed MCF-7 and Jurkat cells to NaAsO_2_. ROS is produced following arsenic exposure (4–7). NAC modulates the alterations of *SOD2* and *CAT* mRNA levels in either MCF-7 or Jurkat cells. Similarly, silymarin or naringenin antioxidants administration was beneficial in the recovery of altered SOD and CAT activity (28). The use of different antioxidants has been found beneficial in various cellular systems exposed to arsenic (24, 25). The NAC (a thiol-containing antioxidant) seems to be a potential antioxidant in inhibiting cellular damage caused by arsenite (32, 33). NAC is regarded not as a direct antioxidant, but it increases the availability of glutathione (GSH) in cells. GSH plays a critical role in maintaining cellular redox homeostasis. Arsenic has the ability to combine with SH groups, thus depleting cellular GSH levels and thiol status (32, 33). The efficient effect of NAC indicates that arsenic changes cellular redox homeostasis by binding with GSH, which is its natural property. Measuring the production of ROS in the MCF-7 and Jurkat cells exposed to NaAsO_2_ in either presence or absence of NAC is recommended for further experiments. The strength of the present findings is needed to support by enzymes activity and the protein levels of the CAT and SOD1 in further experiments.

## Ethical considerations

Ethical issues (Including plagiarism, informed consent, misconduct, data fabrication and/or falsification, double publication and/or submission, redundancy, etc.) have been completely observed by the authors.
